# Overview: Existing Context and the Need to Generate Evidence of the Impact of Large‐Scale Food Fortification on the Prevalence of Anemia

**DOI:** 10.1111/mcn.70093

**Published:** 2025-09-15

**Authors:** Manpreet Chadha, Daniel Lopez de Romana, Helena Pachón, Mandana Arabi

**Affiliations:** ^1^ Nutrition International Ontario Ottawa Canada; ^2^ present address: UNICEF Brussels Belgium; ^3^ Food Fortification Initiative Atlanta Georgia USA

**Keywords:** anemia, food fortification, micronutrient deficiencies, vitamins and minerals

Anemia remains a significant global public health challenge, particularly affecting women and children. According to the World Health Organization (WHO), in 2019, 30% of women aged 15–49 years, and 40% of children under the age of 5 were anemic (WHO 2024). Anemia is associated with adverse maternal and child outcomes, linked to inadequate cognitive and motor development in children, as well as reduced work capacity in adults, thereby impacting a nation's economic progress (WHO 2024).

The World Health Organization endorsed global anemia targets for 2025: to reduce anemia prevalence among women by 50% (WHO 2014). To reach these targets, multiple interventions with evidence of impact must be scaled up and delivered to the populations at large and to specific target population groups.

The etiology of anemia is complex and is known to be multifactorial. Some of these causes are related to nutrient deficiencies while others are non‐nutritional in nature (WHO n.d.). It was previously believed that approximately 50% of all anemias were attributed to iron deficiency, however, it is now known that the contribution of iron deficiency to anemia will significantly vary across contexts (Petry et al. 2016; Chaparro and Suchdev 2019). Factors driving the context‐specific contribution of iron deficiency to anemia include inadequate dietary iron intake, blood loss from parasitic infections, hemorrhage associated with childbirth or menstrual loss, impaired iron absorption, low iron stores at birth, and interactions between iron and other nutrients (WHO 2023a). Besides iron, deficiency in one or more of the micronutrients involved in hemoglobin synthesis or maintenance, including folate, vitamins A, B6 and B12, and riboflavin, could lead to nutritional anemia (Chaparro and Suchdev 2019).

Food fortification is the addition of vitamins and minerals to foods while they are being processed (WHO, & Food and Agriculture Organization of the United Nations 2006). It can effectively deliver multiple nutrients to populations that consume processed foods such as milk, oil, rice, and wheat flour. Fortification reduces nutrient deficiencies and can reduce the prevalence of anemia due to nutrient deficiencies (Keats et al. 2019). In May 2023, the 76th World Health Assembly adopted the resolution on accelerating efforts to prevent micronutrient deficiencies through food fortification, which urges the Member States to establish and scale up food fortification initiatives (WHO 2023b).

This technical supplement aims to shed light on the critical issue of anemia and its potential reduction through large‐scale food fortification (LSFF) programs. We explore the impact of LSFF as a strategy to combat anemia by increasing the intake of key nutrients essential for hemoglobin synthesis. Along with mandating a well‐designed fortification program to ensure benefits to the population, it is also important that the program is sustainable. Countries often find it challenging to determine and effectively implement the steps needed to ensure sustainability. Therefore, structured guidance, tools and resources are needed for stakeholders to establish and implement good and sustainable program design (Rowe and Dodson 2012). A robust design allows for the adaptation of the program to the country context along with keeping in line with global guidance (García‐Casal 2014). Nonetheless, to effectively address anemia, LSFF programs must be designed and implemented to increase micronutrient intake, and in ways that translate into improvements in hemoglobin levels and anemia prevalence. The LSFF programs must consider several factors, including the choice of food vehicle and fortificants, preliminary assessment of micronutrient deficiencies and assessment of health impact, among others (World Health Organization 2016).

The supplement is structured to follow a logical sequence, addressing various aspects of LSFF, from planning and programming to the potential impact on population health, and each paper builds upon the other, offering a comprehensive overview of LSFF as a viable approach to address anemia. The purpose of this supplement is to share insights and guidance on the following four specific topics, which are described in greater detail below:

1. Nutritional anemia reductions due to food fortification among women of childbearing age: a literature review and Bayesian meta‐analysis.
2. Quantifying the potential impact of food fortification on iron intake, hemoglobin concentration and anemia prevalence among women of reproductive age in India.
3. Introducing double fortified salt in social safety net programmes in Madhya Pradesh and Gujarat in India: Success factors, challenges and lessons learned.
4. A blueprint for fortification planning and programing: Lessons learned from an analytical review of existing fortification frameworks.

Women disproportionately bear the burden of anemia (Mildon et al. 2023). While previous systematic reviews or meta‐analyses reviewed the impact of food fortification on nutritional anemia among vulnerable population groups, the review by Dorbu et al. (2025) comprehensively included these references and adds value by reviewing and meta‐analyzing the literature on the impact of fortified wheat flour, maize flour, rice, and oil (singly or combined) on hemoglobin concentration and anemia prevalence solely in women. Furthermore, modeling assessments enable us to predict the expected impacts of LSFF programs on anemia, making it a valuable tool for policy makers and public health practitioners. The modelling assessment by Luo et al. n.d. quantifies the potential impact of iron‐fortified rice, wheat flour, and salt, combined, on iron intake, hemoglobin concentration, and anemia prevalence among women in India. The group simulated scenarios starting with no fortification, followed by intermediate fortification, and finally a maximum fortification scenario. They used a single‐day 24‐h dietary recall survey to estimate mean iron intake under these scenarios and data from the National Family Health Survey 4 (International Institute for Population Sciences IIPS and ICF 2017) to project changes in hemoglobin levels due to increased iron intake, based on established parameters from two meta‐regression analyses.

In addition, the review by Tsang et al. (2024) offers critical components of LSFF programs through the lens of a process evaluation. Understanding the necessary steps and components involved in fortification planning is fundamental to successful planning and implementation of such programs. Lastly, setting the stage for an effective and sustainable food fortification intervention begins with a sound program design (Codling et al. 2015; Martorell et al. 2015; POLICY AND PRACTICE REVIEWS article 2023). It is crucial for countries to identify the essential components to be incorporated in the design of LSFF plans and programs (Karapanou et al. 2024). To provide guidance and support on this issue, this supplement includes a thorough Fortification Blueprint by Darwar et al. (2023). The Blueprint is based on an analytical review of existing fortification frameworks, designed to provide systematic guidance and a repository of tools and resources for fortification program managers and key stakeholders, ensuring the optimal and sustainable design of LSFF programs (Darwar et al. 2023).

The evidence provided in the supplement concludes that food fortification programs have the potential to increase micronutrients intake, increase hemoglobin concentrations and decrease anemia prevalence in women. Nonetheless, strong government buy‐in and technical support along the food fortification pathway and along the supply chain can be key factors in designing and delivering successful food fortification programs.

While LSFF is widely implemented as a public health nutrition strategy, the link between fortification programs and measurable reductions in anemia remains underexplored. This supplement responds to this evidence gap ‐ synthesizing global data through a systematic review and Bayesian meta‐analysis, modeling the potential impact of iron fortification on anemia outcomes in India, presenting lessons from the implementation of double fortified salt in Indian safety net programs, and offering a framework for more effective LSFF planning. Anchoring fortification efforts in research and evidence generation on anemia can help policymakers and program planners in designing effective food fortification strategies to reduce anemia, thereby improving public health impact. By translating research findings into actionable LSFF policies and programs, we can make a substantial impact on reducing the prevalence and detrimental consequences of anemia. We hope that the findings and recommendations in this supplement will help guide LSFF policy and program development, support evidence‐based fortification interventions to reduce anemia and improve maternal and child health outcomes globally.

## Conflicts of Interest

Co‐authors are employed with Nutrition International (NI) and the Food Fortification Initiative (FFI). NI and FFI support evidence generation and assist country leaders to promote, plan, implement, monitor or evaluate food fortification.
